# Antiaging of Cucurbitane Glycosides from Fruits of *Momordica charantia* L.

**DOI:** 10.1155/2018/1538632

**Published:** 2018-03-25

**Authors:** Xueli Cao, Yujuan Sun, Yanfei Lin, Yanjun Pan, Umer Farooq, Lan Xiang, Jianhua Qi

**Affiliations:** College of Pharmaceutical Sciences, Zhejiang University, Yu Hang Tang Road 866, Hangzhou 310058, China

## Abstract

Methanol extracts of *Momordica charantia* L. fruits are extensively studied for their antiaging activities. A new cucurbitane-type triterpenoid (1) and nine other known compounds (2–10) were isolated, and their structures were determined according to their spectroscopic characteristics and chemical derivatization. Biological evaluation was performed on a K6001 yeast bioassay system. The results indicated that all the compounds extended the replicative lifespan of K6001 yeast significantly. Compound 9 was used to investigate the mechanism involved in the increasing of the lifespan. The results indicated that this compound significantly increases the survival rate of yeast under oxidative stress and decreases ROS level. Further study on gene expression analysis showed that compound 9 could reduce the levels of *UTH1* and *SKN7* and increase *SOD1* and *SOD2* gene expression. In addition, it could not extend the lifespan of the yeast mutants of *Uth1*, *Skn7*, *Sod1*, and *Sod2*. These results demonstrate that compound 9 exerts antiaging effects via antioxidative stress and regulation of *UTH1*, *SKN7*, *SOD1*, and *SOD2* yeast gene expression.

## 1. Introduction

Fruits of *Momordica charantia* L. are edible healthy vegetable in Asia and commonly known as bitter melon or bitter gourd because of their bitter taste. Given their nutritional potential, they are used as traditional Chinese herbal medicine to treat several ailments, such as diabetes, constipation, abdominal pain, kidney stones, piles, pneumonia, and improve appetite [1–5]. *M. charantia* contains biologically active phytochemicals, such as polysaccharides, proteins, flavonoids, glycosides, saponins, steroids, alkaloids, essential oils, and triterpenes [5–10]. Many of these phytochemicals exhibit antitumor, anti-inflammatory, immunomodulation, and antidiabetic activities and the ability to reduce oxidative stress [5].

Aging is a dominating risk factor for age-related diseases, including cancer, metabolic disease, cardiovascular disease, and neurodegenerative illnesses [11]. As the aging population is increasing dramatically throughout the world, aging has drawn great attention because of huge expenses for medical care and serious consequences of the related diseases. Interventions that delay aging were found to have a greater effect on the quality of life compared with disease-specific approaches [12]. In our previous studies [13–17], a yeast mutant K6001 was employed in the bioassay system, and ganodermasides A–D, phloridzin, nolinospiroside F, and parishin with significant antiaging potential from natural sources were obtained.

**Figure 1:**
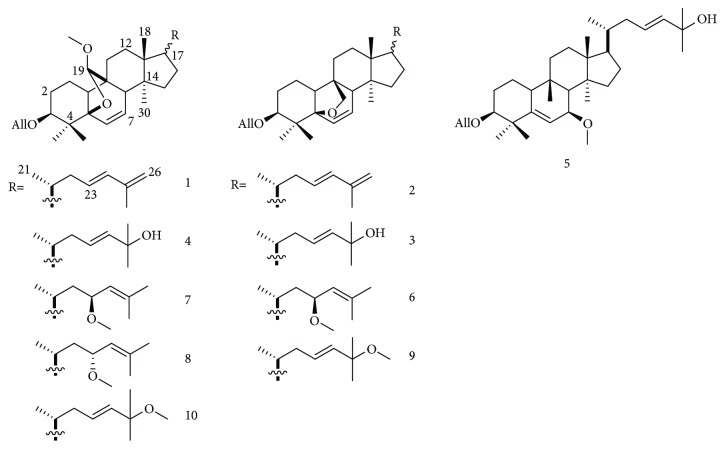
Chemical structures of compounds 1–10.

Basing on the K6001 bioassay system, we isolated one novel cucurbitane glycoside (1) and nine known cucurbitane-type triterpenoids (2–10) from the fruits of *M. charantia* L. (Figure 1). Essential studies on the action mechanism suggested that these cucurbitane glycosides could improve the antioxidative properties of yeasts. The yeast genes of youth 1 (*UTH1*), skinhead-7 (*SKN7*), and superoxide dismutase (*SOD*) may also be involved in the action.

## 2. Material and Methods

### 2.1. General

The chemical reagents used were of HPLC grade and purchased from TEDIA (Rhode Island, USA). The others were of analytical grade and obtained from Sinopharm Chemical Reagent Co. Ltd. (Shanghai, China). The preparative HPLC system was equipped with two ELITE P-230 pumps and an UV detector. Optical rotations were determined on a JASCO P-1030 digital polarimeter. High-resolution ESI-TOF-MS analyses were performed on an Agilent Technologies 6224A Accurate-Mass TOF LC/MS system (Santa Clara, CA, USA). Nuclear magnetic resonance (NMR) spectra were recorded on a Bruker AV III-500 spectrometer (Bruker, Billerica, MA, USA). Column chromatography was performed over the silica gel (200–300 mesh, Yantai Chemical Industry Research Institute, Yantai, China) or reversed phase C18 (Octadecylsilyl, ODS) silica gel (Cosmosil 75C_18_-OPN, Nacalai Tesque, Japan).

### 2.2. Plant Material and Yeast Strains

Fruits of *M. charantia* were purchased from Liangzhu market of Hangzhou, Zhejiang Province, China, 2011. The identity of this plant was confirmed by an associate professor Liurong Chen, and a voucher specimen (number 20110712) was preserved in Zhejiang University Institute of Materia Medica. The yeast strains BY4741 and mutants of *uth1*, *skn7*, *sod1*, and *sod2* with K6001 background are from Prof. Matsuura in Chiba University, and K6001 strain with W303 background is from Prof. Breitenbach in Salzburg University.

### 2.3. Extraction and Isolation

About 1.5 kg (dry weight) of the material was smashed and extracted with methanol (MeOH) for 3 days with shaking at room temperature. The extract was filtered and concentrated to obtain a crude extract (224 g), which was partitioned with ethyl acetate (EtOAc) and water. The active EtOAc layer (30 g) was subjected to a silica gel open column with *n*-hexane/acetone (99 : 1, 98 : 2, 95 : 5, 90 : 10, 80 : 20, 70 : 30, 60 : 40, 50 : 50, 20 : 80, and 0 : 100) and acetone/MeOH (50 : 50 and 0 : 100). The EtOAc layer yielded nine fractions after the combination based on TLC analysis. The eighth active fraction of 4.0 g (13.9 g in total) was subsequently separated by ODS open column with MeOH/H_2_O (60 : 40, 70 : 30, 75 : 25, 80 : 20, 90 : 10, and 100 : 0), and 11 samples were obtained (fr.1–fr.11). The four active samples of fr.6–fr.9 were further separated as follows:

Fr.6 (600 mg) was introduced to an ODS open column with MeOH/H_2_O (70 : 30, 73 : 27, 75 : 25, 77 : 23, 80 : 20, 83 : 17, 85 : 15, and 100 : 0) to yield nine samples (fr.6-1–fr.6-9). Fr.6-6 (72 mg) was further separated by a silica gel open column chromatography with dichloromethane (CH_2_Cl_2_)/MeOH (100 : 0, 99 : 1, 98 : 2, 97.5 : 2.5, 95 : 5, 90 : 10, and 0 : 100), and the sixth active fraction (11.6 mg) was purified by HPLC [C30-UG-5 (*φ*10 × 250 mm, Nomura Chemical), mobile phase: acetonitrile (MeCN)/H_2_O (90 : 10), flow rate: 3 mL/min, and detector: 210 nm] and yielded compound 1 (4.6 mg, *t*_R_ = 33.5 min) and compound 2 (1.0 mg, *t*_R_ = 30.7 min).

Fr.7 (277 mg) was separated in a silica gel open column with chloroform (CHCl_3_)/MeOH (100 : 0, 98 : 2, 95 : 5, 90 : 10, and 0 : 100) and yielded fr.7-1–fr.7-11. Subsequently, fr.7-7 (63 mg) was subjected to ODS open column chromatography with MeOH/H_2_O (70 : 30, 75 : 25, 80 : 20, 90 : 10, and 100 : 0), and the second fraction (37.8 mg) was purified by HPLC [C30-UG-5 (*φ*10 × 250 mm, Nomura Chemical), mobile phase: MeCN/H_2_O (62 : 38), flow rate: 3 mL/min, and detector: 210 nm] and yielded compound 3 (10.6 mg, *t*_R_ = 15.9 min), compound 4 (1.8 mg, *t*_R_ = 17.1 min), and compound 5 (1.9 mg, *t*_R_ = 18.7 min).

Fr.8 (340 mg) was introduced to a silica open column with *n*-hexane/CHCl_3_ (50 : 50, 30 : 70, and 0 : 100) and CHCl_3_/MeOH (97 : 3, 95 : 5, 90 : 10, and 0 : 100) to give fr.8-1–fr.8-9. Then, fr.8-4 (49 mg) was purified by HPLC [CAPCELL PAKC_18_, Shiseido (*φ*10 × 250 mm), mobile phase: MeCN/H_2_O (63 : 37), flow rate: 3 mL/min, and detector: 210 nm] and yielded compound 6 (10.2 mg, *t*_R_ = 20.2 min), compound 7 (4.9 mg, *t*_R_ = 22.9 min), and compound 8 (1.3 mg, *t*_R_ = 32.0 min).

Fr.9 (600 mg) was separated by silica gel open column with CHCl_3_/MeOH (100 : 0, 100 : 1, 100 : 2, 100 : 3, 100 : 5, 90 : 10, and 0 : 100), and fr.9-1–fr.9-8 were obtained. Then, fr.9-4 (332 mg) was further separated by ODS open column with MeOH/H_2_O (90 : 10, 95 : 5, and 100 : 0), and the third fraction (42 mg) was purified by HPLC [CAPCELL PAKC_18_, Shiseido (*φ*10 × 250 mm), mobile phase: MeCN/H_2_O (60 : 40), flow rate: 3 mL/min, and detector: 210 nm] and yielded compound 9 (5.0 mg, *t*_R_ = 28.7 min) and compound 10 (8.0 mg, *t*_R_ = 33.8 min).

#### 2.3.1. Compound 1

White solid; [*α*]_D_^20^-78.6 (*c* 0.2, CH_3_OH); high-resolution ESI-TOF-MS *m*/*z* 653.4029, calculated for C_37_H_58_O_8_Na (M + Na)^+^ 653.4024; data of ^1^H NMR and ^13^C NMR are shown in Table 1.

#### 2.3.2. Charantoside IV (2)

Colorless solid; [*α*]_D_^28^-143.9 (*c* 0.16, CH_3_OH); MS *m*/*z* 623 (M + Na)^+^, C_36_H_56_O_7_Na; ^13^C NMR (125 MHz, pyridine-*d*_5_): *δ* = 15.4, 19.3, 19.3, 19.3, 20.6, 21.5, 24.2, 26.0, 28.0, 28.7, 31.4, 33.8, 37.2, 39.4, 40.5, 40.5, 45.7, 45.9, 49.3, 51.0, 52.7, 63.7, 69.7, 72.9, 73.5, 76.6, 80.5, 85.5, 86.3, 104.3, 115.1, 130.3, 130.4, 134.6, 135.1, and 142.9. The structure was identified based on comparison of MS, ^1^H, and ^13^C NMR data with literature [18].

#### 2.3.3. Momordicoside F_2_ (3)

White solid; [*α*]_D_^20^-101.0 (*c* 0.94, CHCl_3_:CH_3_OH = 1 : 1); MS *m*/*z* 641 (M + Na)^+^, C_36_H_58_O_8_Na; ^13^C NMR (125 MHz, pyridine-*d*_5_): *δ* = 15.4, 19.3, 19.3, 20.6, 21.5, 24.3, 26.0, 28.0, 28.6, 31.3, 31.3, 31.4, 33.8, 37.0, 39.4, 39.9, 40.5, 45.7, 45.8, 49.3, 50.6, 52.7, 63.7, 69.7, 70.1, 72.9, 73.5, 76.6, 80.5, 85.5, 86.3, 104.2, 124.6, 130.4, 134.6, and 142.1. The structure was identified based on comparison of MS, ^1^H, and ^13^C NMR data with literature [19].

#### 2.3.4. Goyaglycoside-b (4)

White solid; [*α*]_D_^20^-100.7 (*c* 0.2, CH_3_OH); MS *m*/*z* 671 (M + Na)^+^, C_37_H_60_O_9_Na; ^13^C NMR (125 MHz, pyridine-*d*_5_): *δ* = 15.3, 19.1, 19.3, 20.4, 21.7, 23.7, 25.3, 27.8, 28.6, 31.3, 31.3, 31.3, 34.3, 37.0, 39.5, 40.0, 42.0, 42.6, 45.7, 48.6, 48.7, 50.8, 58.1, 63.7, 69.7, 70.1, 72.2, 74.2, 77.0, 83.9, 86.0, 102.8, 112.8, 124.7, 132.0, 133.6, and 142.1. The structure was identified based on comparison of MS, ^1^H, and ^13^C NMR data with literature [4].

#### 2.3.5. Karaviloside III (5)

White solid; [*α*]_D_^28^+ 70.9 (*c* 0.1, CH_3_OH); MS *m*/*z* 657 (M + Na)^+^, C_37_H_62_O_8_Na; ^13^C NMR (125 MHz, pyridine-*d*_5_): *δ* = 16.0, 18.5, 19.4, 23.1, 26.3, 28.3, 29.3, 29.4, 29.6, 30.8, 31.3, 31.3, 33.2, 34.8, 35.4, 37.1, 39.8, 40.0, 42.4, 46.7, 48.7, 49.3, 50.6, 56.6, 63.8, 69.7, 70.1, 72.5, 73.9, 76.1, 78.0, 88.3, 105.4, 119.5, 124.2, 142.2, and 148.4. The structure was identified based on comparison of MS, ^1^H, and ^13^C NMR data with literature [20].

#### 2.3.6. Charantoside VI (6)

White solid; [*α*]_D_^28^-67.2 (*c* 0.48, CH_3_OH); MS *m*/*z* 655 (M + Na)^+^, C_37_H_60_O_8_Na; ^13^C NMR (125 MHz, pyridine-*d*_5_): *δ* = 15.2, 18.8, 19.3, 20.3, 20.6, 21.5, 24.2, 26.0, 26.3, 28.0, 29.1, 31.5, 33.8, 34.2, 39.4, 40.5, 43.4, 45.7, 45.9, 49.3, 51.7, 52.7, 55.7, 63.7, 69.7, 72.9, 73.5, 76.6, 76.8, 80.5, 85.6, 86.3, 104.2, 127.8, 130.4, 134.5, and 135.5. The structure was identified based on comparison of MS, ^1^H, and ^13^C NMR data with literature [18].

#### 2.3.7. Charantagenin E (**7**)

Colorless solid; [*α*]_D_^28^-104.3 (*c* 0.11, CH_3_OH); MS *m*/*z* 685 (M + Na)^+^, C_38_H_62_O_9_Na; ^13^C NMR (125 MHz, pyridine-*d*_5_): *δ* = 15.1, 18.8, 19.1, 20.4, 20.4, 21.7, 23.7, 25.3, 26.2, 27.8, 29.0, 31.4, 34.2, 34.2, 39.5, 42.0, 42.6, 43.4, 45.7, 48.5, 48.7, 51.8, 55.7, 58.0, 63.7, 69.7, 72.2, 74.2, 76.9, 77.0, 83.9, 86.0, 102.8, 112.8, 127.8, 132.0, 133.5, and 135.5. The structure was identified according to the comparison among MS, ^1^H, and ^13^C NMR data in literature [21].

#### 2.3.8. Charantoside II (8)

White solid; [*α*]_D_^20^-67.1 (*c* 0.2, CH_3_OH); MS *m*/*z* 685 (M + Na)^+^, C_38_H_62_O_9_Na; ^13^C NMR (125 MHz, pyridine-*d*_5_): *δ* = 15.2, 18.5, 19.1, 19.3, 20.4, 21.6, 23.7, 25.2, 26.2, 27.7, 28.8, 31.4, 33.2, 34.2, 39.5, 42.0, 42.6, 43.7, 45.8, 48.5, 48.7, 51.7, 56.0, 58.0, 63.6, 69.6, 72.2, 74.1, 75.2, 76.9, 84.0, 85.9, 102.9, 112.8, 128.3, 132.0, 133.5, and 134.9. The structure was identified based on comparison of MS, ^1^H, and ^13^C NMR data with literature [18].

#### 2.3.9. Momordicoside G (9)

White solid; [*α*]_D_^28^-90.2 (*c* 1.0, CH_3_OH); MS *m*/*z* 655 (M + Na)^+^, C_37_H_60_O_8_Na; ^13^C NMR (125 MHz, pyridine-*d*_5_): *δ* = 15.5, 19.3, 20.6, 21.5, 24.3, 26.0, 26.5, 26.9, 28.0, 28.6, 31.5, 33.8, 36.7, 39.4, 40.1, 40.5, 45.7, 45.9, 49.3, 50.6, 50.7, 52.7, 63.7, 65.3, 69.7, 72.9, 73.5, 75.3, 76.6, 80.6, 85.5, 86.3, 104.2, 128.8, 130.4, 134.6, and 138.1. The structure was identified based on comparison of MS, ^1^H, and ^13^C NMR data with literature [19].

#### 2.3.10. Goyaglycoside-d (10)

White solid; [*α*]_D_^28^-124.9 (*c* 0.1, CH_3_OH); MS *m*/*z* 685 (M + Na)^+^, C_38_H_62_O_9_Na; ^13^C NMR (125 MHz, pyridine-*d*_5_): *δ* = 15.3, 19.1, 19.3, 20.4, 21.6, 23.7, 25.3, 26.4, 26.9, 27.8, 28.6, 31.3, 34.3, 36.8, 39.5, 40.1, 42.0, 42.6, 45.7, 48.5, 48.7, 50.5, 50.8, 58.1, 63.7, 69.6, 72.2, 74.1, 75.3, 77.0, 83.9, 86.0, 102.8, 112.8, 128.9, 132.0, 133.6, and 138.0. The structure was identified based on comparison of MS, ^1^H, and ^13^C NMR data with literature [4].

### 2.4. Acid Hydrolysis and Sugar Analysis of Compound 1

The absolute configuration of sugar moiety of compound 1 was determined according to a previously reported method [22, 23]. Briefly, compound 1 (0.5 mg) in anhydrous 2.0 M HCl in MeOH (1 mL) was heated at 80°C with reflux for 4 h. The reaction solution was evaporated and partitioned between chloroform and water. The residue of aqueous layer was heated with 0.5 mg L-cysteine methyl ester in pyridine (200 *μ*L) at 60°C for 1 h; then, *o*-tolyl isothiocyanate dissolved in 100 *μ*L pyridine (7 mg/mL) was added to the reaction mixture and further reacted at 60°C for 1 h. After that, the reaction mixture was dried and analyzed by LC/HRESIMS with the following conditions: Agilent Extend C18 column (3.5 *μ*m, 3.0 × 100 mm); DAD detection, 210 nm; *t* = 0 min CH_3_OH/H_2_O/formic acid (30 : 70 : 0.1), *t* = 15 min CH_3_OH/H_2_O/formic acid (60 : 40 : 0.1); and flow rate: 0.45 mL/min.

The allose thiocarbamate standards were prepared in the same procedure. Given that L-allose is limitedly available, the retention time of L-allose thiocarbamate derivative was obtained by reacting D-allose with D-cysteine methyl ester. The basis of this approach is the fact that the *t*_R_ values of D- and L-enantiomers are reversed when D-cysteine methyl ester is used [22].

### 2.5. Lifespan Assay

The bioassay method was performed as described in a previous study [13]. Briefly, K6001 or mutants with K6001 background were grown on a YPGalactose medium consisting of 3% galactose, 2% hipolypeptone, and 1% yeast extract or on a YPGlucose medium containing 2% glucose instead of galactose. Agar plates were prepared by adding 2% agar to the medium. For screening, the K6001 yeast strain was first incubated in the galactose medium for 24 h with shaking and then centrifuged. The yeast pellet was washed with PBS three times. The cells were then diluted and counted using a hemocytometer, and approximately 4000 cells were plated on glucose agar plates containing different concentrations of samples. The plates were stored in an incubator at 28°C. After 48 h, the yeast cells in the plates were observed with a microscope. For each plate, 40 colonies were selected randomly, and the number of their daughter cells was counted and analyzed.

### 2.6. Antioxidative Stress Method

Antioxidative stress assay was performed as previously described with minor modification [16]. BY4741 yeast was inoculated in 5 mL of YPGlucose medium and cultured at 28°C with shaking for 24 h. The yeast cells at 0.1 OD_600_ were transferred in 20 mL of new YPGlucose medium and incubated with compound 9 at 1 and 3 *μ*M or resveratrol (Res, positive control) at 10 *μ*M for 12 h.

For the first method, 5 *μ*L aliquot after double dilution from each group was dropped in the same YPGlucose agar plate mixed with 9 mM H_2_O_2_, and the plate was incubated at 28°C for 4 days. The growth rates of the yeast cells in different groups were compared and photographed.

Another antioxidative stress assay was used to validate the accuracy of the experiment. Approximately 200 cells mixed with the test samples were spreaded on YPGlucose agar plates with or without 5 mM H_2_O_2_ and cultured at 28°C for 48 h. The survival rates of the sample groups were counted and compared with those of the control group.

### 2.7. Determination of ROS Level in Yeast

The ROS assay procedure was the same with a previous study [17]. BY4741 yeast cells were cultured as described in the experiment above and incubated with compound 9 at 1 or 3 *μ*M for 23 h. Changes in intracellular ROS levels of the yeast were determined using an ROS assay kit (Beyotime, Jiangsu, China) and a fluorescent plate reader (Spectra Max M2, Molecular Devices, San Francisco, CA, USA). A total of 1 mL of cultured broth was obtained, treated with 10 *μ*M DCFH-DA at 28°C in dark, and then shaken by vortexing at 160 rpm at 15 min intervals for 1 h. The yeast cells were subsequently washed with PBS, and their DCF fluorescence was measured by a fluorescent plate reader at excitation and emission wavelengths of 488 and 525 nm, respectively.

### 2.8. Real-Time Quantitative PCR Analysis

BY4741 yeast cells were cultured in glucose medium following the addition of 0, 1, 3 *μ*M compound 9. RNA was extracted from yeast cells in the exponential phase through the hot-phenol method. Reverse transcription was performed using a cloned AMV first-strand cDNA synthesis kit (Invitrogen, California, USA) with oligo (dT) adaptor primers and 5 *μ*g of yeast total RNA. Real-time PCR was performed using the CFX96-Touch (Bio-Rad, Hercules, USA) and SYBR Premix EX Taq™ (TaKaRa, Otsu, Japan). Thermal cycling parameters for *UTH1* and *SKN7*: 40 cycles, 94°C for 15 s, 55.4°C for 15 s, and 68°C for 20 s; for *SOD1* and *SOD2*: 40 cycles, 94°C for 15 s, 60°C for 25 s, and 72°C for 10 s. Primers used were as follows: for *UTH1*, sense 5′-CGC CTC TTC CTC TT-3′ and antisense 5′-ACC ATC GGA AGG TTG TTC AG-3′; for *SKN7*, sense 5′-AGT TGT CAG CGA CGG TCT TT-3′ and antisense 5'-GCT GTG GCA CCA TCT AGG TT-3′; for *SOD1* sense 5′-CAC CAT TTT CGT CCG TCT TT-3′ and antisense 5′-TGG TTG TGT CTC TGC TGG TC-3′; for *SOD2*, sense 5′-CTC CGG TCA AAT CAA CGA AT-3′ and antisense 5′-CCT TGG CCA GAA GAT CTG AG-3′; for *TUB1*, sense 5′-CCA AGG GCT ATT TAC GTG GA-3′ and antisense 5′-GGT GTA ATG GCC TCT TGC AT-3′. The amount of *UTH1*, *SKN7*, *SOD1*, and *SOD2* was normalized to that of *TUB1.*

### 2.9. Statistical Analysis

One-way analysis of variance was performed using GraphPad Prism biostatistics software (San Diego, CA, USA) to analyze the data. Significant differences were compared by two-tailed multiple *t*-tests with Student–Newman–Keuls test. Data were expressed as means ± SEM of triplicate experiments. A *P* < 0.05 was considered statistically significant.

## 3. Results and Discussion

### 3.1. Structure Elucidation of Compound 1

**Figure 2:**
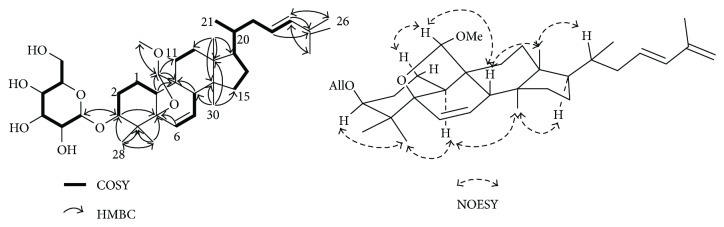
Gross structure of compound 1 with ^1^H-^1^H COSY, selected HMBC, and NOESY correlations.

Compound 1 has the molecular formula C_37_H_58_O_8_ as determined by HR-ESIMS measurement. The ^1^H NMR data showed six methyl groups at *δ*_H_ 0.83 (3H, s), 0.90 (3H, s), 0.91 (3H, s), 0.95 (3H, d, *J* = 5.6 Hz), 1.47 (3H, s), and 1.92 (3H, s), along with six olefinic protons at *δ*_H_ 4.96 (1H, s), 5.10 (1H, s), 5.63 (1H, dd, *J* = 3.6, 9.7 Hz), 5.76 (1H, m), 6.18 (1H, dd, *J* = 2.10, 9.7 Hz), and 6.32 (1H, d, *J* = 15.3 Hz). Several multiple peaks at *δ*_H_ 3.94–4.76 and the signal of an anomeric proton [*δ*_H_ 5.52 (1H, d, *J* = 7.8 Hz)] indicated the existence of a sugar moiety. The ^13^C NMR data revealed the presence of 37 carbon signals. With the combined signals of ^13^C NMR and DEPT, the 37 carbon signals were attributed to six olefinic carbons (*δ*_C_ 115.1, 130.4, 132.0, 133.6, 135.1, and 143.0), one anomeric carbon (*δ*_C_ 102.8), one oxygenated quaternary carbon (*δ*_C_ 86.0), six oxymethines (*δ*_C_ 69.7, 72.2, 74.2, 77.0, 83.9, and 112.8), one oxymethylene (*δ*_C_ 63.7), one methoxy group (*δ*_C_ 58.1), four quaternary sp^3^ carbons (*δ*_C_ 39.5, 45.8, 48.6, and 48.7), four methines (*δ*_C_ 37.3, 42.1, 42.7, and 51.2), seven methylenes (*δ*_C_ 19.2, 23.7, 27.8, 28.7, 31.3, 34.3, and 40.5), and six methyl groups (*δ*_C_ 15.3, 19.3, 19.4, 20.4, 21.7, and 25.3). Detailed analysis of the ^1^H-^1^H COSY spectra led to the determination of the partial structures depicted by the bonds (Figure 2, in bold bonds). In the HMBC spectrum, these partial structures were connected to yield the following gross structures: H-3 to C-5; H-6 to C-5; H-8 to C-9; H-10 to C-9; CH_3_-18 to C-12, C-13, C-14, and C-17; H-19 to C-9, C-10, C-11, and −OCH_3_; CH_3_-28 to C-3, C-4, and C-29; CH_3_-29 to C-4, C-5, and C-28; CH_3_-30 to C-8, C-13, C-14, and C-15; H-23 to C-25; H-24 to C-25, C-26, and C-27; H-26 to C-24; CH_3_-27 to C-24; and −OCH_3_ to C-19. The signals of H-3 to C-1′ of allose and anomeric proton H-1′ of allose to C-3 in the HMBC indicated the location of the sugar moiety (Figure 2). The *β* anomeric configuration of allose was determined from its coupling constant *J* (7.8 Hz) of anomeric protons (*δ*_H_ 5.52). The absolute configuration of the sugar moiety was further confirmed by the degradation of compound 1 and through the comparison of the retention time of its aldose thiocarbamate derivative (*t*_R_ = 9.287 min) with those of the following aldose thiocarbamate standards: L-cysteine-D-allose (*t*_R_ = 9.127 min) and D-cysteine-D-allose (*t*_R_ = 7.460 min).

The relative stereochemistry of compound 1 was deduced by nuclear overhauser enhancement spectroscopy (NOESY) analysis. As shown in Figure 2, the broad singlet signal of H-3 appeared at *δ*_H_ 3.73 thereby suggested the *α* configuration of this proton. The NOESY correlations of major cross-peaks of H-3*α*/Me-28, Me-28/H-10, H-10/Me-30, and Me-30/H-17 indicated the *α*-orientation of these protons. The correlations between H-1*β*/H-19, H-19/H-8, H-8/Me-18, and Me-18/H-20 suggested the *β*-orientation of these groups. The double bond at C-23 and C-24 was elucidated by COSY correlations, whereas the transgeometry was determined from the coupling constant, *J*_23-24_ = 15.3 Hz.

The above evidence suggested that compound 1 was structurally similar to (19*R*, 23*E*)-5*β*, 19-epoxy-19-methoxycucurbita-6, 23-25-trien-3*β*-ol 3-O-*β*-D-allopyranoside (Figure 1).

### 3.2. Identification of the Known Compounds

Compounds 2–10 (Figure 1) were identified by comparing their spectroscopic data with those in literature.

### 3.3. Antiaging Activity in K6001 Yeast Strain

**Figure 3:**
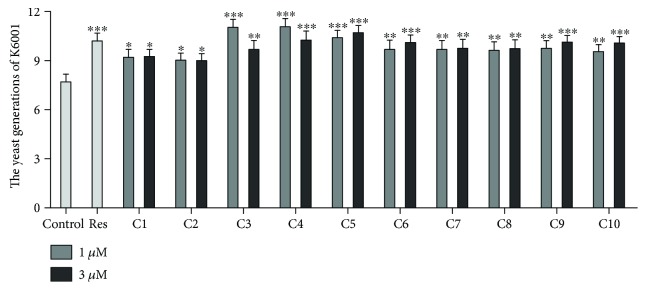
Effect of compounds 1–10 on the replicative lifespan of K6001 yeast strain. The average lifespan of K6001 was as follows: control (7.70 ± 0.48); Res at 10 *μ*M (10.20 ± 0.42^∗∗∗^); compound 1 at 1 *μ*M (9.20 ± 0.49^∗^) and at 3 *μ*M (9.25 ± 0.42^∗^); compound 2 at 1 *μ*M (9.03 ± 0.43^∗^) and at 3 *μ*M (9.00 ± 0.42^∗^); compound 3 at 1 *μ*M (11.03 ± 0.53^∗∗∗^) and at 3 *μ*M (9.68 ± 0.55^∗∗^); compound 4 at 1 *μ*M (11.08 ± 0.50^∗∗∗^) and at 3 *μ*M (10.25 ± 0.56^∗∗∗^); compound 5 at 1 *μ*M (10.40 ± 0.45^∗∗∗^) and at 3 *μ*M (10.70 ± 0.45^∗∗∗^); compound 6 at 1 *μ*M (9.68 ± 0.57^∗∗^) and at 3 *μ*M (10.10 ± 0.46^∗∗∗^); compound 7 at 1 *μ*M (9.68 ± 0.55^∗∗^) and at 3 *μ*M (9.75 ± 0.55^∗∗^); compound 8 at 1 *μ*M (9.63 ± 0.52^∗∗^) and at 3 *μ*M (9.73 ± 0.55^∗∗^); compound 9 at 1 *μ*M (9.75 ± 0.47^∗∗^) and at 3 *μ*M (10.13 ± 0.41^∗∗∗^); and compound 10 at 1 *μ*M (9.55 ± 0.42^∗∗^) and at 3 *μ*M (10.08 ± 0.39^∗∗∗^) (^∗^*P* < 0.05, ^∗∗^*P* < 0.01, and ^∗∗∗^*P* < 0.001, compared with the control).

All isolated cucurbitane triterpenoids (1–10**)** were tested for antiaging activity through the K6001 bioassay method at different optimum concentrations. All the compounds at 1 and 3 *μ*M extended the replicative lifespan of K6001 significantly (Figure 3), demonstrating that the cucurbitane triterpenoids isolated from *M. charantia* L. fruits have antiaging effect in yeast.

### 3.4. Compound 9 Improves the Oxidative Resistance and Decreases ROS Production of Yeast

**Figure 4:**
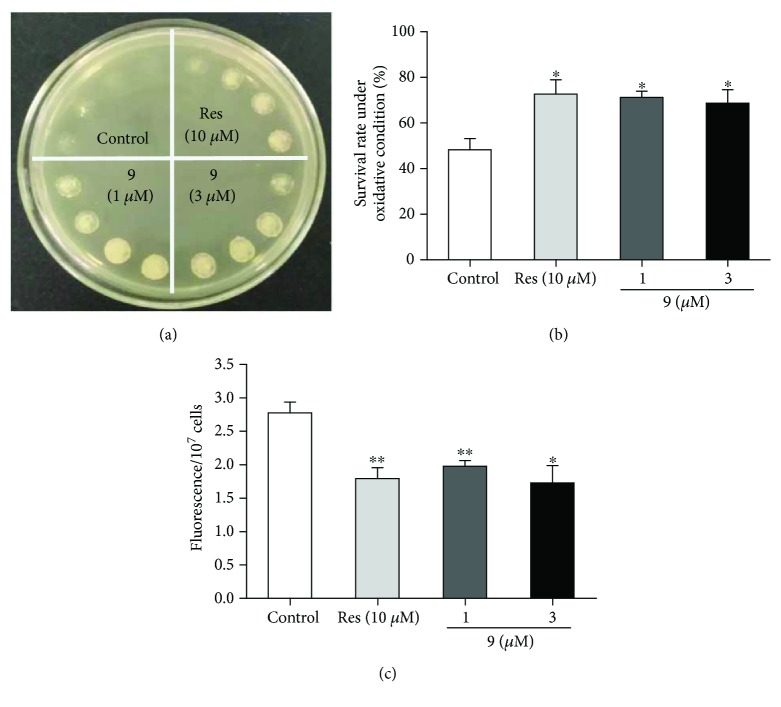
Effect of compound 9 on the antioxidative ability of yeast cells and ROS level of yeast. (a) BY4741 yeast cells in the control group and compound 9-treated groups were dropped in the same YPGlucose agar plate mixed with 9 mM H_2_O_2_. After four days, the growth of yeast cells in different groups was photographed. (b) Effect of compound 9 on the survival rates of yeast under oxidative stress condition. Control, 48.31 ± 4.94; Res, 72.74 ± 6.19^∗^; compound 9 at 1 *μ*M, 71.21 ± 2.75^∗^and at 3 *μ*M, 68.73 ± 5.89^∗^. The experiment was conducted at least thrice. Vertical bars represent the mean ± SEM of three assays (^∗^*P* < 0.05). (c) The change of ROS level of yeast after administration compound 9 at 1, 3 *μ*M. Control, 2.77 ± 0.15^∗∗^; Res, 1.79 ± 0.15^∗∗^; compound 9 at 1 *μ*M, 1.98 ± 0.08^∗∗^, and at 3 *μ*M, 1.73 ± 0.22^∗^. Vertical bars represent the mean ± SEM of 6 repeats (^∗^*P* < 0.05 and ^∗∗^*P* < 0.01, compared with the control).

Studies on mechanism of action were conducted with compound 9 because of its abundance and good activity. Oxidative stress is one of the primary causes of aging, as indicated in various model organisms [24]. Therefore, the effect of compound 9 on the oxidative resistance of yeast was first tested. The growth of yeast cells was inhibited at 9 mM H_2_O_2_, whereas incubation with compound 9 at 1 or 3 *μ*M remitted the inhibition (Figure 4(a)). The effect was further confirmed in another assay. As shown in Figure 4(b), the survival rate of the control group was 48.31% ± 4.94%, whereas that in the experimental groups increased to 71.21% ± 2.75% (compound 9 at 1 *μ*M, *P* < 0.5) and 68.73% ± 5.89% (compound 9 at 3 *μ*M, *P* < 0.5). The experiments indicated that compound 9 enhances the oxidative resistance of yeast cells. Furthermore, we detected the ROS level of yeast after administration compound 9 at 1 and 3 *μ*M. As we expected, the ROS level of yeast in the resveratrol and compound 9 groups were significantly decreased compared with the control group (Figure 4(c), *P* < 0.01, *P* < 0.01, and *P* < 0.05), respectively. These results suggested that compound 9 extended the replicative lifespan via inhibition of oxidative stress.

### 3.5. Compound 9 Extends Yeast Lifespan via Modification of UTH1, SKN7, SOD1, and SOD2 Gene Expression

**Figure 5:**
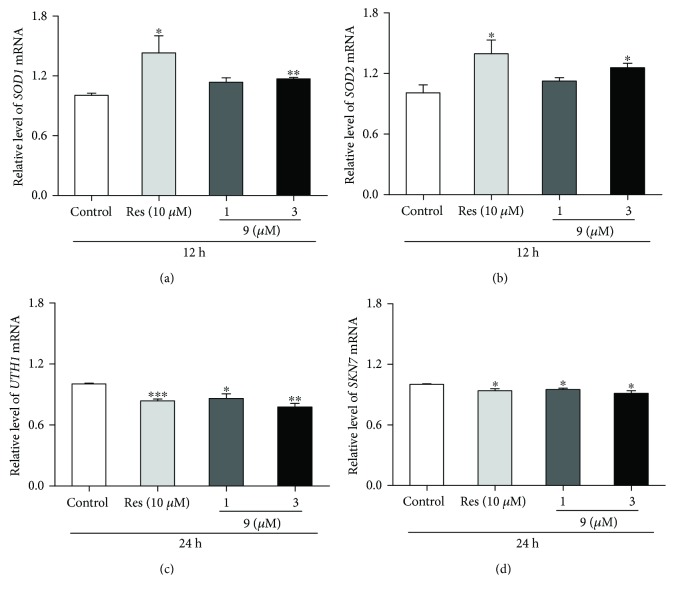
Effects of compound 9 on *SOD1* (a), *SOD2* (b), *UTH1* (c), and *SKN7* (d) yeast gene expression. The gene levels of BY4741 yeast cells were tested after treated with compound 9 at 1 and 3 *μ*M. Compound 9 significantly increased *SOD1* and *SOD2* yeast gene level at 12 h and inhibited *UTH1* and *SKN7* yeast gene expression at 24 h. Amounts of the mRNA above were normalized to that of *TUB1*. The results were displayed as mean ± SEM for three independent experiments (^∗^*P* < 0.05, ^∗∗^*P* < 0.01, and ^∗∗∗^*P* < 0.001, compared with the control group).

It is well known that antioxidative stress is one of mechanisms of action for antiaging. *UTH1* gene essentially takes part in oxidative stress regulation, and deletion of *UTH1* gene will lead to extend the replicative lifespan of yeast [25]. *SKN7* is upstream gene and is a stress response transcription factor in *Saccharomyces cerevisiae* [26]. Superoxide dismutases (SOD) are major ROS scavenging enzymes and can convert superoxide anion to hydrogen peroxide [24]. Real-time PCR analysis was performed to examine the molecular mechanism of compound 9-mediated lifespan extension. The significant gene expression reduction or increase of *UTH1*, *SKN7*, *SOD1*, and *SOD2* was observed in the compound 9 treatment groups (Figure 5). These results suggested that compound 9 produced antiaging effect via regulation *UTH1*, *SKN7*, *SOD1*, and *SOD2* yeast gene expression.

### 3.6. Antiaging Effects of Compound 9 Diminished in Uth1, Skn7, Sod1, and Sod2 Mutations with K6001 Background

**Figure 6:**
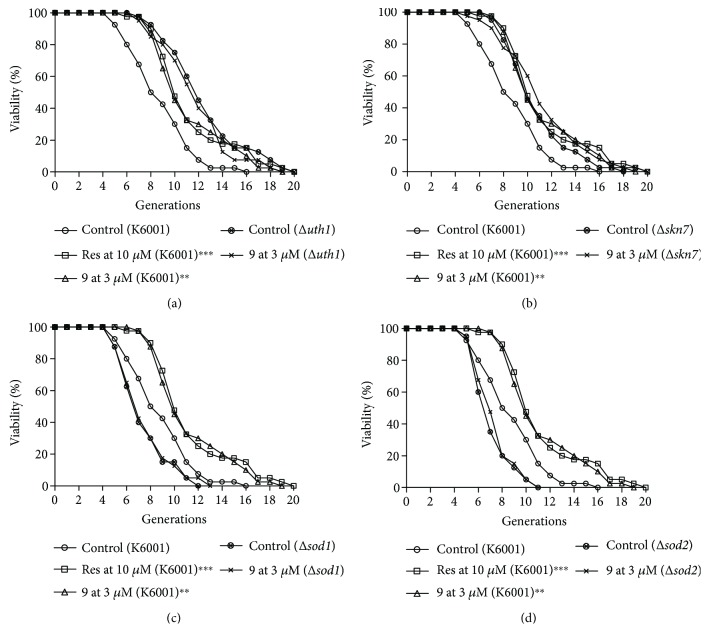
Effect of compound 9 on the replicative lifespan of *uth1* (a), *skn7* (b), *sod1* (c), and *sod2* (d) mutants. The average lifespan of K6001 in the control group was 7.93 ± 0.41; Res at 10 *μ*M, 10.45 ± 0.52^∗∗∗^; and compound 9 at 3 *μ*M, 10.33 ± 0.49^∗∗^. (a) The average lifespan of Δ*uth1* in the control group was 11.60 ± 0.51 and compound 9 at 3 *μ*M, 10.95 ± 0.46. (b) The average lifespan of Δ*skn7* in the control group was 9.88 ± 0.41 and compound 9 at 3 *μ*M, 10.40 ± 0.53. (c) The average lifespan of Δ*sod1* in the control group was 6.55 ± 0.32 and compound 9 at 3 *μ*M, 6.65 ± 0.34. (d) The average lifespan of Δ*sod2* in the control group was 6.28 ± 0.25 and compound 9 at 3 *μ*M, 6.50 ± 0.25 (^∗∗^*P* < 0.01 and ^∗∗∗^*P* < 0.001).

To investigate the role of these genes in the antiaging activity of compound 9, we used the mutants of *uth1*, *skn7*, *sod1*, and *sod2*. As shown in Figure 6, compound 9 at 3 *μ*M did not affect the replicative lifespan of *uth1* (Figure 6(a)) or *skn7* mutants (Figure 6(b)), neither of *sod1* (Figure 6(c)) or *sod2* mutants (Figure 6(d)). These results were further indicated that these four genes were involved in the mechanism of action of compound 9.

## 4. Conclusions

A novel cucurbitane-type triterpenoid and nine known compounds were isolated and identified from the fruits of *M. charantia*. All the compounds showed antiaging effect in yeast. The antiaging activities of these cucurbitane-type triterpenoids depended on their antioxidative ability and the regulation of the *UTH1*, *SKN7*, *SOD1*, and *SOD2* yeast genes. Apart from being one of the most well-known vegetables and frequently used as a traditional medicine because of its health benefits, *M. charantia* has potential as an antiaging functional food.

## Figures and Tables

**Table 1:** ^1^H-NMR (500 MHz) and ^13^C-NMR (125 MHz) data of compound 1 in pyridine-*d*_5_.

Position	Compound 1	
	*δ*_H_ (*J* in Hz)	*δ*_C_
1*α*	1.46	19.2
1*β*	1.91	—
2*α*	1.75	27.8
2*β*	2.17	—
3*α*	3.73 (br s)	83.9
4	—	39.5
5	—	86.0
6	6.18 (dd, 2.0, 9.7)	133.6
7	5.63 (dd, 3.6, 9.7)	132.0
8*β*	3.15 (br s)	42.7
9	—	48.6
10*α*	2.48 (dd, 5.6, 12.7)	42.1
11*α*	1.75	23.7
11*β*	1.68	—
12*α*	1.60	31.3
12*β*	1.55	—
13	—	45.8
14	—	48.7
15*α*	1.31	34.3
15*β*	1.31	—
16*α*	1.94	28.7
16*β*	1.32	—
17*α*	1.50	51.2
18	0.91 (s)	15.3
19	4.91 (s)	112.8
20	1.50	37.3
21	0.95 (d, 5.6)	19.4
22*α*	1.83	40.5
22*β*	2.32	—
23	5.76 (m)	130.4
24	6.32 (d, 15.3)	135.1
25	—	143.0
26*α*	4.96 (s)	115.1
26*β*	5.10 (s)	—
27	1.92 (s)	19.3
28	0.83 (s)	25.3
29	1.47 (s)	21.7
30	0.90 (s)	20.4
−OCH_3_	3.52 (s)	58.1
1´	5.52 (d, 7.8)	102.8
2´	3.94 (dt, 2.6, 7.6)	74.2
3´	4.76 (d, 2.8)	72.2
4´	4.23 (td, 2.8, 9.3)	69.7
5´	4.50 (m)	77.0
6´*α*	4.43 (m)	63.7
6´*β*	4.57 (m)	—

## References

[1] Nagarani G., Abirami A., Siddhuraju P. (2014). Food prospects and nutraceutical attributes of *Momordica* species: a potential tropical bioresources – a review. *Food Science and Human Wellness*.

[2] Xu X., Shan B., Liao C. H., Xie J. H., Wen P. W., Shi J. Y. (2015). Anti-diabetic properties of *Momordica charantia* L. polysaccharide in alloxan-induced diabetic mice. *International Journal of Biological Macromolecules*.

[3] Raman A., Lau C. (1996). Anti-diabetic properties and phytochemistry of *Momordica charantia* L. (Cucurbitaceae). *Phytomedicine*.

[4] Murakami T., Emoto A., Matsuda H., Yoshikawa M. (2001). Medicinal foodstuffs. XXI. Structures of new cucurbitane-type triterpene glycosides, goyaglycosides-a, -b, -c, -d, -e, -f, -g, and -h, and new oleanane-type triterpene saponins, goyasaponins I, II, and III, from the fresh fruit of Japanese Momordica charantia L.. *Chemical and Pharmaceutical Bulletin*.

[5] Grover J. K., Yadav S. P. (2004). Pharmacological actions and potential uses of *Momordica charantia*: a review. *Journal of Ethnopharmacology*.

[6] Horax R., Hettiarachchy N., Chen P. (2014). Characteristics and functionality enhancement by glycosylation of bitter melon (*Momordica charantia*) seed protein. *Journal of Food Science*.

[7] Li Z.-J., Chen J.-C., Deng Y.-Y. (2015). Two new cucurbitane triterpenoids from immature fruits of *Momordica charantia*. *Helvetica Chimica Acta*.

[8] Zhang B., Xie C., Wei Y., Li J., Yang X. (2015). Purification and characterisation of an antifungal protein, MCha-Pr, from the intercellular fluid of bitter gourd (*Momordica charantia*) leaves. *Protein Expression and Purification*.

[9] Kenny O., Smyth T. J., Hewage C. M., Brunton N. P. (2013). Antioxidant properties and quantitative UPLC-MS analysis of phenolic compounds from extracts of fenugreek (*Trigonella foenum-graecum*) seeds and bitter melon (*Momordica charantia*) fruit. *Food Chemistry*.

[10] Zhang F., Lin L., Xie J. (2016). A mini-review of chemical and biological properties of polysaccharides from *Momordica charantia*. *International Journal of Biological Macromolecules*.

[11] Armanios M., de Cabo R., Mannick J., Partridge L., van Deursen J., Villeda S. (2015). Translational strategies in aging and age-related disease. *Nature Medicine*.

[12] Kaeberlein M., Rabinovitch P. S., Martin G. M. (2015). Healthy aging: the ultimate preventative medicine. *Science*.

[13] Weng Y., Xiang L., Matsuura A., Zhang Y., Huang Q., Qi J. (2010). Ganodermasides A and B, two novel anti-aging ergosterols from spores of a medicinal mushroom *Ganoderma lucidum* on yeast via *UTH1* gene. *Bioorganic & Medicinal Chemistry*.

[14] Weng Y., Lu J., Xiang L. (2011). Ganodermasides C and D, two new anti-aging ergosterols from spores of the medicinal mushroom *Ganoderma lucidum*. *Bioscience, Biotechnology, and Biochemistry*.

[15] Xiang L., Sun K., Lu J. (2011). Anti-aging effects of phloridzin, an apple polyphenol, on yeast *via* the SOD and Sir2 genes. *Bioscience, Biotechnology, and Biochemistry*.

[16] Sun K., Cao S., Pei L., Matsuura A., Xiang L., Qi J. (2013). A steroidal saponin from *Ophiopogon japonicus* extends the lifespan of yeast via the pathway involved in *SOD* and *UTH1*. *International Journal of Molecular Sciences*.

[17] Lin Y., Sun Y., Weng Y., Matsuura A., Xiang L., Qi J. (2016). Parishin from *Gastrodia elata* extends the lifespan of yeast via regulation of Sir2/Uth1/TOR signaling pathway. *Oxidative Medicine and Cellular Longevity*.

[18] Akihisa T., Higo N., Tokuda H. (2007). Cucurbitane-type triterpenoids from the fruits of *Momordica charantia* and their cancer chemopreventive effects. *Journal of Natural Products*.

[19] Okabe H., Miyahara Y., Yamauchi T. (1982). Studies on the Constituents of Momordica charantia L. III. Characterization of New Cucurbitacin Glycosides of the Immature Fruits. Structures of Momordicosides G, F_1_, F_2_ and I. *Chemical and Pharmaceutical Bulletin*.

[20] Nakamura S., Murakami T., Nakamura J., Kobayashi H., Matsuda H., Yoshikawa M. (2006). Structures of new cucurbitane-type triterpenes and glycosides, karavilagenins and karavilosides, from the dried fruit of *Momordica charantia* L. in Sri Lanka. *Chemical & Pharmaceutical Bulletin*.

[21] Wang X., Sun W., Cao J., Qu H., Bi X., Zhao Y. (2012). Structures of new triterpenoids and cytotoxicity activities of the isolated major compounds from the fruit of *Momordica charantia* L.. *Journal of Agricultural and Food Chemistry*.

[22] Tanaka T., Nakashima T., Ueda T., Tomii K., Kouno I. (2007). Facile discrimination of aldose enantiomers by reversed-phase HPLC. *Chemical & Pharmaceutical Bulletin*.

[23] Berrué F., McCulloch M. W. B., Boland P. (2012). Isolation of steroidal glycosides from the Caribbean sponge *Pandaros acanthifolium*. *Journal of Natural Products*.

[24] Kong Y., Trabucco S. E., Zhang H. (2014). Oxidative stress, mitochondrial dysfunction and the mitochondria theory of aging. *Interdisciplinary Topics in Gerontology*.

[25] Camougrand N., Kissova I., Velours G., Manon S. (2004). Uth1p: a yeast mitochondrial protein at the crossroads of stress, degradation and cell death. *FEMS Yeast Research*.

[26] Basso V., Znaidi S., Lagage V. (2017). The two-component response regulator Skn7 belongs to a network of transcription factors regulating morphogenesis in *Candida albicans* and independently limits morphogenesis-induced ROS accumulation. *Molecular Microbiology*.

